# Gene Co-Expression Network Analysis Provides Novel Insights into Myostatin Regulation at Three Different Mouse Developmental Timepoints

**DOI:** 10.1371/journal.pone.0117607

**Published:** 2015-02-19

**Authors:** Xuerong Yang, James E. Koltes, Carissa A. Park, Daiwen Chen, James M. Reecy

**Affiliations:** 1 Animal Nutrition Institute, Sichuan Agricultural University, Ya’an, Sichuan, China; 2 Department of Animal Science, Iowa State University, Ames, Iowa, United States of America; Harvard Medical School, UNITED STATES

## Abstract

Myostatin (*Mstn*) knockout mice exhibit large increases in skeletal muscle mass. However, relatively few of the genes that mediate or modify MSTN effects are known. In this study, we performed co-expression network analysis using whole transcriptome microarray data from MSTN-null and wild-type mice to identify genes involved in important biological processes and pathways related to skeletal muscle and adipose development. Genes differentially expressed between wild-type and MSTN-null mice were further analyzed for shared DNA motifs using DREME. Differentially expressed genes were identified at 13.5 d.p.c. during primary myogenesis and at d35 during postnatal muscle development, but not at 17.5 d.p.c. during secondary myogenesis. In total, 283 and 2034 genes were differentially expressed at 13.5 d.p.c. and d35, respectively. Over-represented transcription factor binding sites in differentially expressed genes included SMAD3, SP1, ZFP187, and PLAGL1. The use of regulatory (RIF) and phenotypic (PIF) impact factor and differential hubbing co-expression analyses identified both known and potentially novel regulators of skeletal muscle growth, including *Apobec2*, *Atp2a2*, and *Mmp13* at d35 and *Sox2*, *Tmsb4x*, and *Vdac1* at 13.5 d.p.c. Among the genes with the highest PIF scores were many fiber type specifying genes. The use of RIF, PIF, and differential hubbing analyses identified both known and potentially novel regulators of muscle development. These results provide new details of how MSTN may mediate transcriptional regulation as well as insight into novel regulators of MSTN signal transduction that merit further study regarding their physiological roles in muscle and adipose development.

## Introduction

Myostatin (MSTN), also known as GDF8, is a transforming growth factor beta (TGFβ) superfamily member that negatively regulates skeletal muscle growth in various species [1–3]. Previous studies have demonstrated that MSTN blockade can functionally improve dystrophic muscle [4] and that MSTN can also play an important role in adipose development [5]. To better understand the molecular mechanisms by which MSTN controls muscle growth and development, microarray data from MSTN-null and wild-type mice were used for gene co-expression network analysis during key timepoints in skeletal muscle development. These timepoints included primary myogenesis at 13.5 days post coitus (d.p.c.), secondary myogenesis at 17.5 d.p.c., and postnatal muscle growth at 35 days after birth (d35) [6–8].

In previous studies, the partial correlation and information theory (PCIT) approach and the regulatory impact factor (RIF) algorithm have been used to evaluate the regulatory functions of transcription factors (TFs) on gene expression [9–11]. The PCIT algorithm can be used to determine the differential hubbing (DH; [9]) of targets to putative regulatory genes. A regulator that exhibits DH across treatments will have drastically higher connectivity in one treatment compared to the others. The RIF algorithm identifies potential regulators that are “differentially wired” (i.e., differentially correlated) as a function of treatment (*Mstn* genotype, in the context of this study). Two methods for calculating RIF assign different weights to the target gene’s level of expression. The RIF1 score also ranks genes based on their phenotypic impact factor (PIF; a score that combines overall expression and differential expression). RIF1 scores place more emphasis on target transcripts highly differentially wired due to treatment, while RIF2 scores identify regulators whose expression tends to predict changes in expression of target transcripts that are differentially expressed (DE) between treatments. Previous studies evaluating RIF scores demonstrate that genes with relatively stable expression levels in different conditions (i.e., genes not differentially expressed) can still play important roles in biological processes in a coordinated manner. But these studies have limitations: first, these analyses mainly considered DE genes, which were quite limited and may not be representative of all the changes that take place in response to an experimental treatment. Second, only genes with known regulatory functions (i.e., transcription factors) were evaluated in previous studies. Without considering all genes, it is difficult to discover novel regulators of skeletal muscle growth.

In this study, we applied modified versions of the PCIT [12] and RIF algorithms [10,11] to evaluate more than 34,000 annotated genes from a whole microarray dataset as potential regulators of muscle and adipose development in the presence or absence of functional myostatin. We consider the RIF method modified because all genes were tested as possible regulators using RIF scores, whereas previous studies using this methodology considered only TFs as possible regulators. The PCIT method utilized in this anlaysis used the same methodology but was computationally enhanced to complete the analysis in considerably less time than the original algorithm [12]. We calculated RIF scores that contrasted *Mstn* genotypes at each developmental timepoint to identify potential regulators of *Mstn* or pathways influenced by MSTN signal transduction. Furthermore, DE genes were compared across *Mstn* genotypes for differences in the enrichment of TF binding sites, biological processes, and pathways related to MSTN function.

## Materials and Methods

### Ethics Statement

This study was carried out in strict accordance with the recommendations in the Animal Guide of the United States Department of Agriculture. The protocol was approved by the Institutional Animal Care and Use committee of Iowa State University (Protocol Number: 11-05-6013-M).

### Animals and sample collection

Animals were reared, sacrificed by CO_2_ asphyxiation (embryonic timepoints) or cervical dislocation (d35), and tissues were collected from whole embryos (13.5 d.p.c.), hindquarters (17.5 d.p.c.), or pectoralis muscle (d35). The pectoralis muscle was chosen at day 35 because it is the muscle with the greatest change in muscle mass due to *Mstn* deletion and had a high level of myostatin expression as described in the original paper characterizing myostatin function in skeletal muscle [2]. After collection, muscles were flash-frozen in liquid nitrogen as previously described [13].

### RNA isolation and target preparation

The frozen tissues from whole embryo or hindquarter only were immersed in RLT lysis buffer (Qiagen, Valencia, CA) and homogenized for 45 seconds, then centrifuged at 3600g for 10 min to pellet cell debris. Total RNA was isolated using the RNase Midi Kit (Qiagen) with on-column DNase digestion according to manufacturer's protocol. Isolation from pectoralis muscle was conducted as described [13]. The concentration of isolated RNA was evaluated based on absorbance at 260 nm and the quality was determined by agarose gel electrophoresis. For microarray analysis, 10 μg of RNA from each of three individual animals was pooled to give five pools representing 15 indviduals for each genotype/timepoint combination. A final concentration of 1 μg/ml was yielded from the pooled RNA, the quality of which was reconfirmed using the 2100 Bioanalyzer (Agilent Technologies, Palo Alto, CA). Microarray target preparation, hybridization, and scanning were conducted as described [13].

### Microarray processing

Target RNA was hybridized to both chip A and chip B of the GeneChip Mouse Expression Set 430 (MOE430; Affymetrix, Santa Clara, CA), which includes over 45,000 probe sets and 39,000 transcripts and variants, containing over 34,000 well-annotated mouse genes. Robust multi-array analysis (RMA) was performed to normalize the raw data [14] by using R/affy package [15]. All of the microarray data is available at NCBI GEO under accession number GSE63154.

### Statistical analysis

The normalized gene expression data were used for further analysis. To detect differentially expressed genes in each genotype, expression data with five replicates were analyzed as a completely randomized design by using R/limma package [16]. Because the tissues are different across timepoints, the analysis was performed separately for each timepoint. The model used for analysis was, where y_ij_ is the normalized gene expression, μ is the overall mean, G_i_ is the ith genotype, and ε_ij_ is the error for the jth replicate within the ith genotype. DE genes were identified at a false discovery rate (FDR) <0.05 using the q-value package from Bioconductor [17].

### RIF analysis and differential hubbing

Using all of the Affy ProbesetIDs after excluding the control IDs and housekeeping genes, we applied a modified partial correlation and information theory approach (PCIT) [10,12] to compute the co-expression correlation between each gene pair in both MSTN-null and wild-type conditions. Co-expression analyses were done only within timepoint since tissue types were not identical across different developmental stages. As described in Reverter et al. [11], we calculated the regulatory impact factors (RIF), which compute the differential wiring from the difference in co-expression correlation of each pair of genes. A key difference in this analysis is that we used all genes for the RIF analysis to try to identify novel regulators of muscle growth in response to lack of functional MSTN. Differential hubbing calculations were conducted using a Perl script that captured only the significant partial correlations identified by the PCIT algorithm. Correlates were filtered to include only those genes with |r| ≥ 0.90 to ensure that only strong correlations were captured, given the small sample size and large amount of data. The concept of RIF, PIF, and differential hubbing can be challenging to understand in the context of biological processes. Hudson et al. [9] provide a nice description of how these co-expression methods can be used to understand the genetic regulation of muscle mass.

### Annotation and pathway analysis

Annotation of ProbesetIDs is based on the annotation file from the Affymetrix website (http://www.affymetrix.com), while the IDs without annotation were annotated using Biomart (Ensembl, http://www.ensembl.org/biomart/martview/c56c1fc10cb311e0b5fe1c05dbd3f217) and DAVID [18]. Differentially expressed genes and genes co-correlated to top RIF were used to identify over-represented pathways across different developmental timepoints in web-based DAVID, while the significant regulators of these gene lists were analyzed in Sub-Network Enrichment Analysis (SNEA) via Pathway Studio [19].

### Discovering significantly over-represented motifs using DREME

Among those genes up- and downregulated between genotypes as well as co-expressed with top RIF regulators, we applied a motif discovery method for non-redundant, statistically significant, and discriminative motifs using Discriminative Regular Expression Motif Elicitation (DREME) [20] implemented in iPlant (http://www.iplantcollaborative.org). We used 1,500 bp upstream flanking sequence in FASTA format. Generally, we ran the analysis using the default settings. First, the sequences of genes of interest from up- and downregulated groups were used for positive input sequences, while the negative sequences were the sequences of well-annotated genes from both MOE430A and MOE430B chips after excluding those genes of interest. Second, we chose 3 as the minimum width of core motif and 8 as the maximum. Third, 0.05 was chosen as the motif e-value cutoff, where the e-value is the motif statistic p-value times the number of candidate motifs tested, and the counts created after removing sites that matched motifs found previously were used to calculate the p-value.

To evaluate the discovered motifs, we used TOMTOM [21] implemented in MEME Suite (http://meme.nbcr.net/meme/intro.html) to compare the motifs with the database of known transcription factor motifs in JASPAR (JASPAR_CORE_2009.meme; 476 motifs) [22] and UniProbe (uniprobe_mouse.meme; 386 motifs) [23] using default settings with the significance threshold 0.05.

## Results

### Differentially expressed genes

Table 1 and Table 2 present the top 20 DE genes ranked by significance at 13.5 d.p.c. and d35, respectively. The DE genes were identified using R/limma (S1 Table) and a significance threshold of FDR < 0.05. At 13.5 d.p.c., 283 genes were DE, 17 of which were downregulated and 266 of which were upregulated in WT. Surprisingly, we did not identify any differentially expressed genes (FDR < 0.05) at 17.5 d.p.c. At d35, 2034 genes were DE, 928 of which were downregulated and 1106 of which were upregulated in wild-type mice.

**Table 1:** Top 20 WT/null differentially expressed genes ranked by significance at 13.5 d.p.c., q < 0.05*.

ProbesetID	Fold Change^^^	q-Value	Change Direction	Gene Symbol	Gene Description
1419356_at	1.94	1.44E-05	Up	Klf7	Kruppel-like factor 7 (ubiquitous)
1456257_at	0.62	0.0118	Down	Fam126b	family with sequence similarity 126, member B
1431686_a_at	1.22	0.0130	Up	Gmfb	glia maturation factor, beta
1418170_a_at	1.26	0.0130	Up	Zcchc14	zinc finger, CCHC domain containing 14
1427353_at	1.27	0.0130	Up	Clasp1	CLIP associating protein 1
1422528_a_at	1.28	0.0130	Up	Zfp36l1	zinc finger protein 36, C3H type-like 1
1456398_at	1.29	0.0130	Up	TUG1	taurine upregulated gene 1
1451730_at	1.29	0.0130	Up	Zfp62	zinc finger protein 62
1431326_a_at	1.29	0.0130	Up	Tmod2	tropomodulin 2
1449118_at	1.34	0.0130	Up	Dbt	dihydrolipoamide branched chain transacylase E2
1436746_at	1.41	0.0130	Up	Wnk1	WNK lysine deficient protein kinase 1
1426756_at	1.41	0.0130	Up	Galnt2	UDP-N-acetyl-alpha-D-galactosamine:polypeptide N-acetylgalactosaminyltransferase 2
1419038_a_at	1.58	0.0130	Up	Csnk2a1	casein kinase 2, alpha 1 polypeptide
1431239_at	1.18	0.0135	Up	Nono	non-POU-domain-containing, octamer binding protein
1424398_at	1.26	0.0135	Up	Dhx36	DEAH (Asp-Glu-Ala-His) box polypeptide 36
1426777_a_at	1.43	0.0139	Up	Wasl	Wiskott-Aldrich syndrome-like (human)
1417623_at	1.18	0.0142	Up	Slc12a2	solute carrier family 12, member 2
1455886_at	1.26	0.0142	Up	Cbl	Casitas B-lineage lymphoma
1445199_at	1.22	0.0149	Up	PATZ1	POZ (BTB) and AT hook containing zinc finger 1
1457513_at	1.22	0.0149	Up	Fam40a	family with sequence similarity 40, member A

*For multiple probesetIDs with the same annotation, the probesetID with smallest q-value was saved.

^^^Fold changes are presented as WT/null. Fold change > 1 indicates higher expression in the WT compared to the null. Fold change < 1 indicates higher expression in the null genotype compared to the WT.

**Table 2:** Top 20 WT/null differentially expressed genes ranked by significance at d35, q < 0.05*.

ProbesetID	Fold Change^^^	q-Value	Change Direction	Gene Symbol	Gene Description
1451203_at	2.28	5.65E-06	Up	Mb	Myoglobin
1416551_at	3.74	5.65E-06	Up	Atp2a2	ATPase, Ca++ transporting, cardiac muscle, slow twitch 2
1427768_s_at	3.80	1.07E-05	Up	Myl3	myosin, light polypeptide 3
1449997_at	4.74	1.47E-05	Up	Tpm3	tropomyosin 3, gamma
1448152_at	0.40	1.67E-05	Down	Igf2	insulin-like growth factor 2
1419606_a_at	5.50	1.67E-05	Up	Tnnt1	troponin T1, skeletal, slow
1455450_at	1.76	1.92E-05	Up	Ptpn3	protein tyrosine phosphatase, non-receptor type 3
1424831_at	0.63	2.28E-05	Down	Cpne2	copine II
1448554_s_at	4.36	2.28E-05	Up	Myh6	myosin, heavy polypeptide 6, cardiac muscle, alpha
				Myh7	myosin, heavy polypeptide 7, cardiac muscle, beta
1450813_a_at	4.30	5.98E-05	Up	Tnni1	troponin I, skeletal, slow 1
1418370_at	4.93	5.98E-05	Up	Tnnc1	troponin C, cardiac/slow skeletal
1417959_at	0.64	6.51E-05	Down	Pdlim7	PDZ and LIM domain 7
1417673_at	1.77	6.51E-05	Up	Grb14	growth factor receptor bound protein 14
1437025_at	0.49	7.72E-05	Down	Cd28	CD28 antigen
1425153_at	1.82	7.72E-05	Up	Myh2	myosin, heavy polypeptide 2, skeletal muscle, adult
1448394_at	5.08	8.62E-05	Up	Myl2	myosin, light polypeptide 2, regulatory, cardiac, slow
1419145_at	2.06	9.75E-05	Up	Smtnl1	smoothelin-like 1
1424937_at	1.58	9.98E-05	Up	Plin5	perilipin 5
1422813_at	0.60	1.01E-04	Down	Cacng1	calcium channel, voltage-dependent, gamma subunit 1
1418865_at	0.60	1.06E-04	Down	Zfp385a	zinc finger protein 385A

*For multiple probesetIDs with the same annotation, the probesetID with smallest q-value was saved.

^^^Fold changes are presented as WT/null. Fold change > 1 indicates higher expression in the WT compared to the null. Fold change < 1 indicates higher expression in the null genotype compared to the WT.

### DAVID annotation and Pathway Studio analysis of DE genes at 13.5 d.p.c. and d35

Pathway analysis results from DAVID and Pathway Studio 9 are listed in S2 and S3 Tables. The 283 genes DE at 13.5 d.p.c. were significantly enriched for 46 Gene Ontology (GO) terms [24] and several PANTHER pathway terms and SP_PIR_KEYWORDS [18]. Most of the 13.5 d.p.c. DE genes were involved in the regulation of transcription, RNA processing, negative regulation of cellular biosynthetic process and transcription, nucleotide binding, transcription repressor activity, and WNT signaling pathway. For the 2034 genes DE at d35, 58 GO terms, 19 SP_PIR_KEYWORDS, and several KEGG and PANTHER pathway terms were over represented. The DE genes at d35 were largely involved in muscle organ/tissue development, fatty acid metabolism, and mitochondria. Pathway Studio results at d35 identified 100 significantly over-represented regulator-target relationships (p < 0.05); PPARG, LEP, calmodulin, and GH1 were linked to the largest numbers of differentially expressed genes. MEF2A, MEF2C, creatine kinase, and UCP2 were also significantly enriched in the regulator-target relationships.

### Over-represented DNA motifs in differentially expressed genes

**Fig 1:**
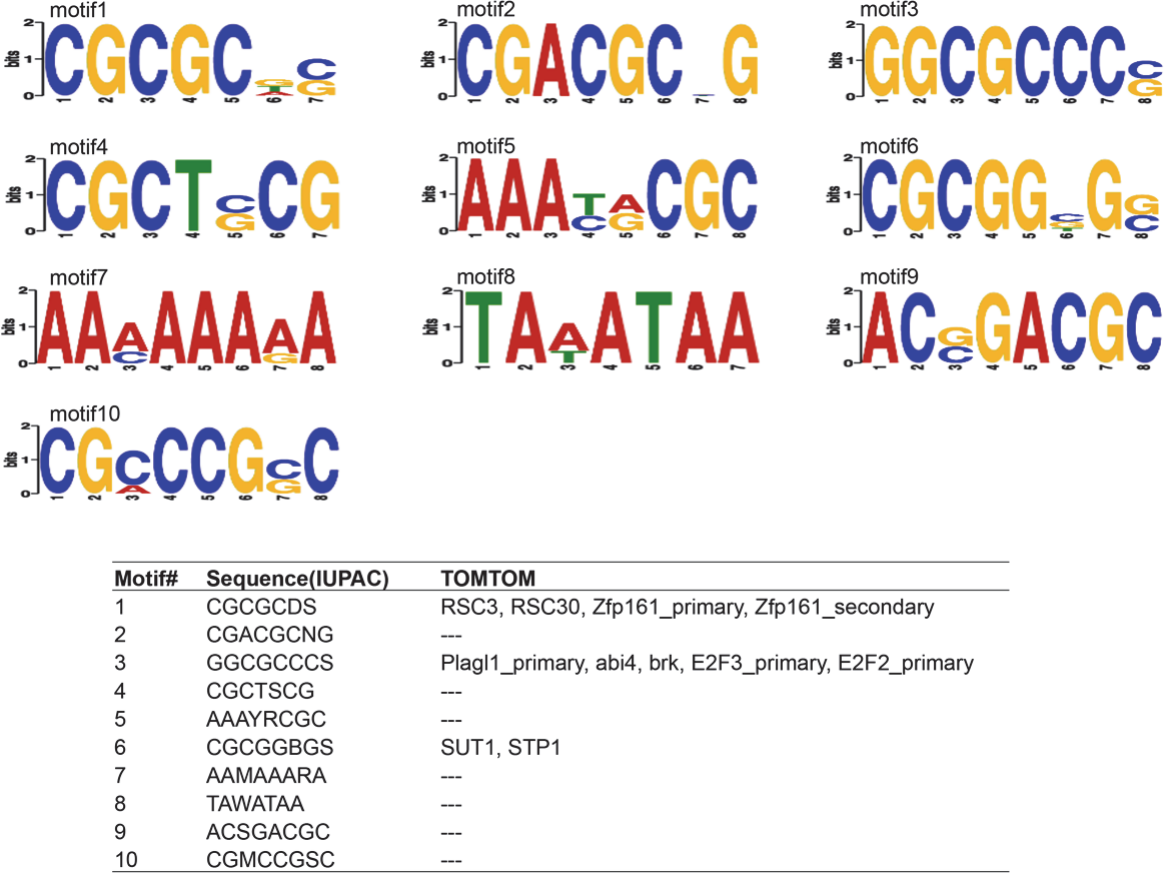
Over-represented motifs in genes upregulated in WT at 13.5 d.p.c.

**Fig 2:**
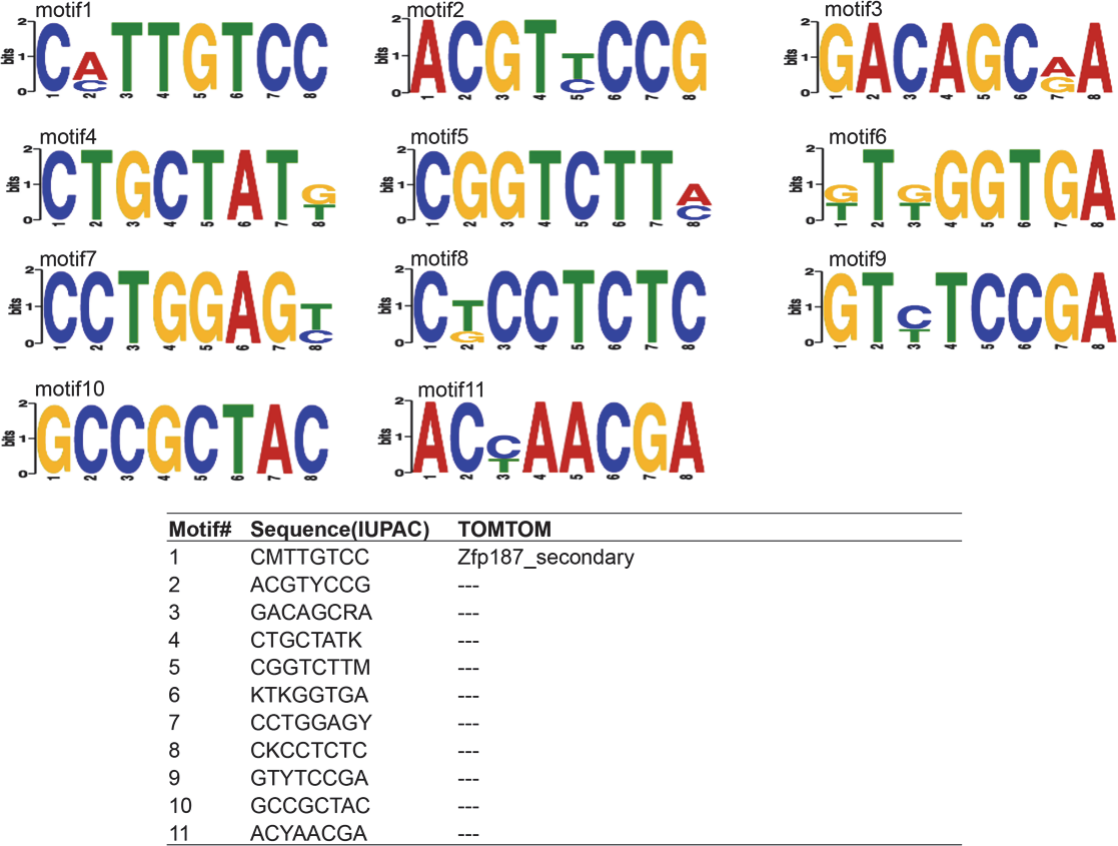
Over-represented motifs in genes upregulated in MSTN-null at 13.5 d.p.c.

Fig. 1 and Fig. 2 illustrate the over-represented motifs present in the promoters of DE genes at 13.5 d.p.c. (E-values < 0.05). Ten and eleven over-represented motifs were identified in DE genes upregulated in WT and MSTN–null genotypes, respectively. Over-represented motifs at d35 are listed in Figures A and B in S1 File. At this timepoint, nine and ten over-represented motifs were identified in DE genes upregulated in WT and MSTN-null genotypes, respectively. Similar to the 13.5 d.p.c. timepoint, enriched motifs were identified in zinc-finger protein binding sites. Moreover, the SMAD3 binding site was identified as an over-represented motif in DE genes upregulated in the MSTN-null genotype, and the SP1 binding site was found to be the sixth most significant motif in DE genes upregulated in the WT.

### Regulatory impact factor analysis

**Fig 3:**
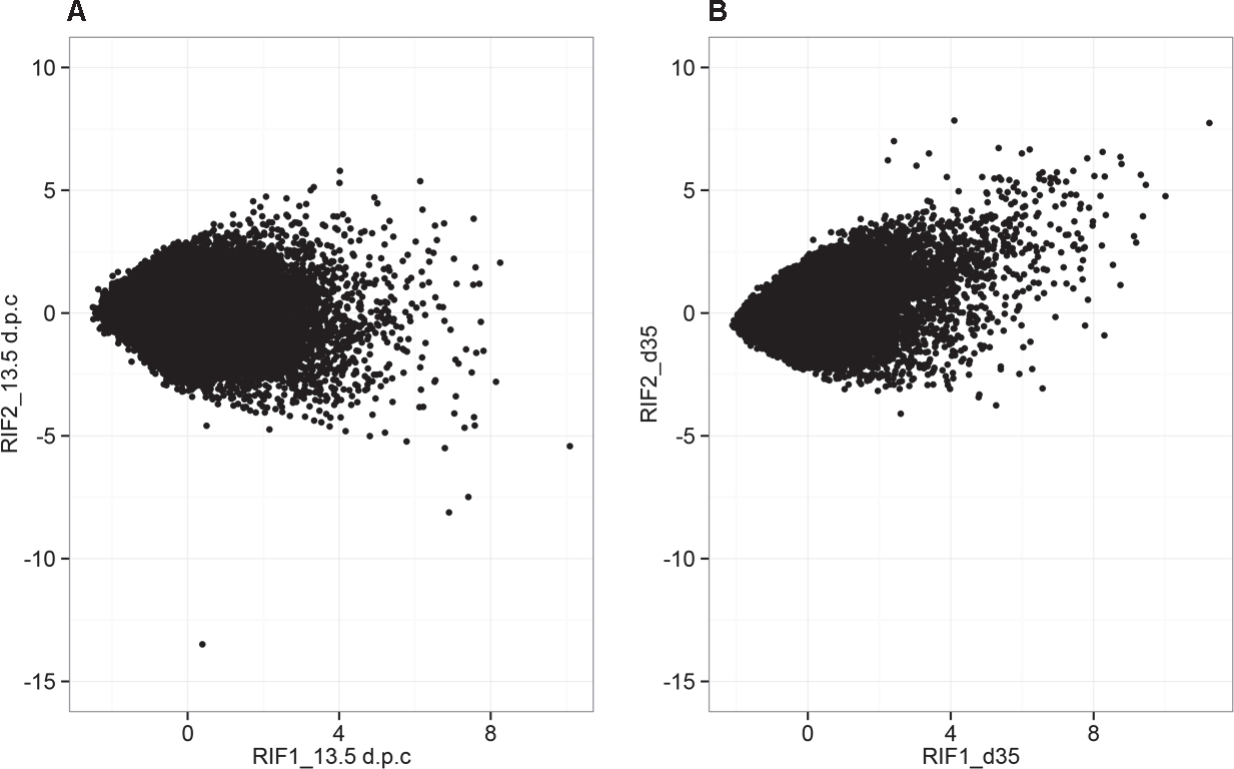
Relationship between RIF1 and RIF2 at 13.5 d.p.c. and d35.

Tables 3–6 list the top ten most extreme RIF scores, ranked by Z-score, at 13.5 d.p.c. and d35. All RIF scores are provided in S4 Table. Fig. 3 shows the relationship between RIF1 and RIF2 scores for the 13.5 d.p.c. and d35 timepoints. The correlation coefficient (r) between RIF1 and RIF2 was very close to zero at 13.5 d.p.c. (r = -0.09) and moderately positive at d35 (r = 0.45). At 13.5 d.p.c., *Rps29* and *Beta-s* exhibited the top two RIF1 Z-scores (10.09 and 8.25, respectively). At d35 after birth, *Tnni2* and *Tpm1* exhibited the top two RIF1 Z-scores (11.24 and 10.01, respectively). At 13.5 d.p.c., *Cox6b1* and *Rpl13a* exhibited the top two RIF2 Z-scores (5.79 and 5.37, respectively), and at d35, *Atp2a2* and *Tnni2* exhibited the top two RIF2 Z-scores (7.84 and 7.74, respectively). Several genes were identified with top-ten RIF scores at both timepoints (i.e., *Beta-s*, *GM10420*, *March-2*). In addition, *Tnni2* was identified in the top ten at d35 for both RIF1 and RIF2. Target genes highly connected to *Tnni2* (Pearson correlation coefficient based on PCIT, |r| ≥ 0.9) were further investigated using ontology enrichment analysis to identify potential pathways involved in muscle development. Genes highly correlated with *Tnni2* in wild-type mice were enriched in the KEGG pathways viral myocarditis, immune/cancer, and proteolysis (adj.P, Benjamini < 0.1; S5 Table). However, no KEGG terms were significantly enriched in *Tnni2* correlates in MSTN-null mice.

**Table 3:** Top 10 RIF1 (wild type minus null) at 13.5 d.p.c.

ProbesetID	RIF1	RIF2	Gene Symbol	Gene Description
(1) Top Positive RIF1				
1415833_x_at	10.09	-5.42	Rps29	ribosomal protein S29
1417184_s_at	8.25	2.05	Beta-s, Hbb-b1, Hbb-b2	hemoglobin subunit beta-1-like
				hemoglobin, beta adult major chain
				hemoglobin, beta adult minor chain
1416054_at	8.14	-2.80	Rps5	ribosomal protein S5
1416546_a_at	7.81	-1.54	Rpl6	ribosomal protein L6
1417451_a_at	7.74	-0.36	Ppia	peptidylprolyl isomerase A
(2) Top Negative RIF1				
1415924_at	-2.50	0.23	Tnp1	transition protein 1
1416055_at	-2.50	0.24	Amy2a1, Amy2a2	amylase 2a1, amylase 2a2, amylase 2a3
			Amy2a3, Amy2a4, Amy2a5	amylase 2a4, amylase 2a5
1418855_at	-2.49	-0.25	Lce1l	late cornified envelope 1L
1420239_x_at	-2.45	0.21	March-2	membrane-associated ring finger (C3HC4) 2
1419792_at	-2.44	0.00	Kif20b	kinesin family member 20B

**Table 4:** Top 10 RIF2 (wild type minus null) at 13.5 d.p.c.

ProbesetID	RIF2	RIF1	Gene Symbol	Gene Description
(1) Top Positive RIF2				
1416565_at	5.79	7.00	Cox6b1	cytochrome c oxidase, subunit VIb polypeptide 1
1417608_a_at	5.37	6.14	Rpl13a	ribosomal protein L13A
1423111_at	5.30	4.01	Atp5a1	ATP synthase, H+ transporting, mitochondrial F1 complex
				alpha subunit 1
1436992_x_at	5.13	3.33	Vdac1	voltage-dependent anion channel 1
1417771_a_at	5.00	3.25	Psmc6	proteasome (prosome, macropain) 26S subunit, ATPase, 6
(2) Top Negative RIF2				
1435873_a_at	-13.49	0.39	Gm11478, Rpl13a	60S ribosomal protein L13a-like, ribosomal protein L13A
			Zfp526	zinc finger protein 526
1416141_a_at	-8.12	6.90	Rps6, Rps6-ps4	ribosomal protein S6, ribosomal protein S6, pseudogene 4
1431765_a_at	-7.49	7.41	Gm10420, Gm5978, Gm6139	predicted gene 10420, predicted gene 5978, predicted gene 6139
			Gm8225, Gm8842, Rps2	predicted gene 8225, predicted gene 8842, ribosomal protein S2
1415906_at	-5.50	6.79	Tmsb4x	thymosin, beta 4, X chromosome
1415833_x_at	-5.42	10.09	Rps29	ribosomal protein S29

**Table 5:** Top 10 RIF1 (wild type minus null) at d35.

ProbesetID	RIF1	RIF2	Gene Symbol	Gene Description
(1) Top Positive RIF1				
1416889_at	11.24	-0.92	Tnni2	troponin I, skeletal, fast 2
1423049_a_at	10.01	-0.50	Tpm1	tropomyosin 1, alpha
1416478_a_at	9.46	1.25	Mdh2	malate dehydrogenase 2, NAD (mitochondrial)
1416921_x_at	9.38	1.40	Aldoa, Aldoart1	aldolase A, fructose-bisphosphate, aldolase 1 A retrogene 1
1417184_s_at	9.32	2.05	Beta-s	hemoglobin subunit beta-1-like
			Hbb-b1	hemoglobin, beta adult major chain
			Hbb-b2	hemoglobin, beta adult minor chain
(2) Top Negative RIF1				
1417051_at	-2.11	-0.04	Pcdh8	protocadherin 8
1420239_x_at	-2.08	0.21	March2	membrane-associated ring finger (C3HC4) 2
1416744_at	-2.07	0.20	Uap1	UDP-N-acetylglucosamine pyrophosphorylase 1
1416967_at	-2.07	-1.26	Sox2	SRY-box containing gene 2
1417256_at	-2.03	0.08	Mmp13	matrix metallopeptidase 13

**Table 6:** Top 10 RIF2 (wild type minus null) at d35.

ProbesetID	RIF2	RIF1	Gene Symbol	Gene Description
(1) Top Positive RIF2				
1416551_at	7.84	4.1	Atp2a2	ATPase, Ca++ transporting, cardiac muscle, slow twitch 2
1416889_at	7.74	11.24	Tnni2	troponin I, skeletal, fast 2
1419606_a_at	7.00	2.41	Tnnt1	troponin T1, skeletal, slow
1416850_s_at	6.72	5.34	Cisd1	CDGSH iron sulfur domain 1
1417889_at	6.66	6.21	Apobec2	apolipoprotein B mRNA editing enzyme, catalytic polypeptide 2
(2) Top Negative RIF2				
1423255_at	-4.1	2.6	Atp6v1g1	ATPase, H+ transporting, lysosomal V1 subunit G1
1422156_a_at	-3.76	5.27	Gm10420, Gm5978, Gm6139, Gm8841, Gm8842, Rps2	predicted gene 1042, predicted gene 597, predicted gene 6139, predicted gene 8841, predicted gene 8842, ribosomal protein S2
1426731_at	-3.43	4.78	Des	desmin
1422613_a_at	-3.32	4.8	Gm11362, Rpl7a	predicted gene 11362, ribosomal protein L7A
1418996_a_at	-3.17	1.96	Lyrm5	LYR motif containing 5

### Differential hubbing

**Fig 4:**
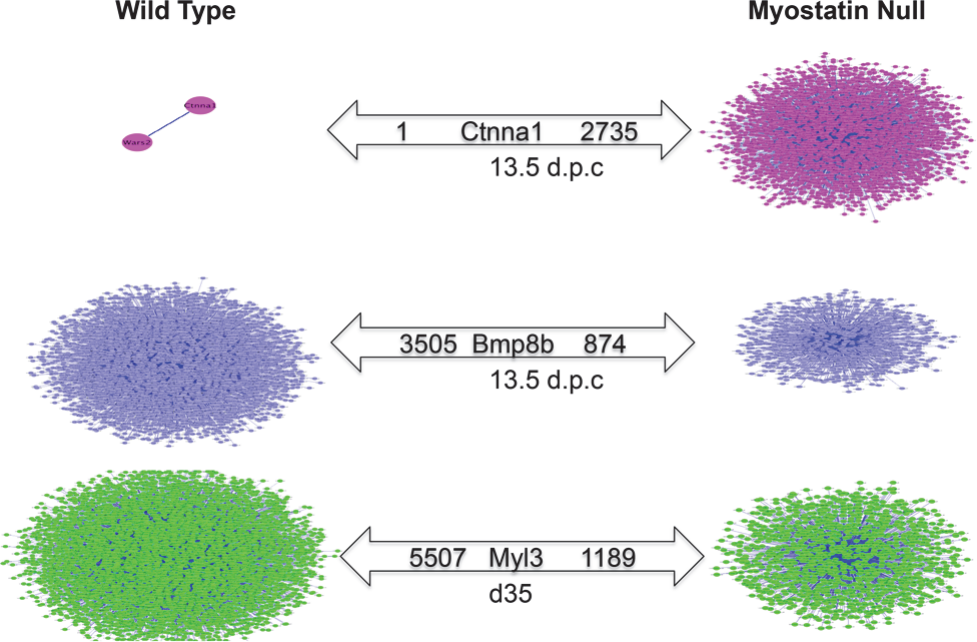
Differential wiring of co-correlated genes between WT and MSTN-null mice (|r| ≥ 0.9).

**Fig 5:**
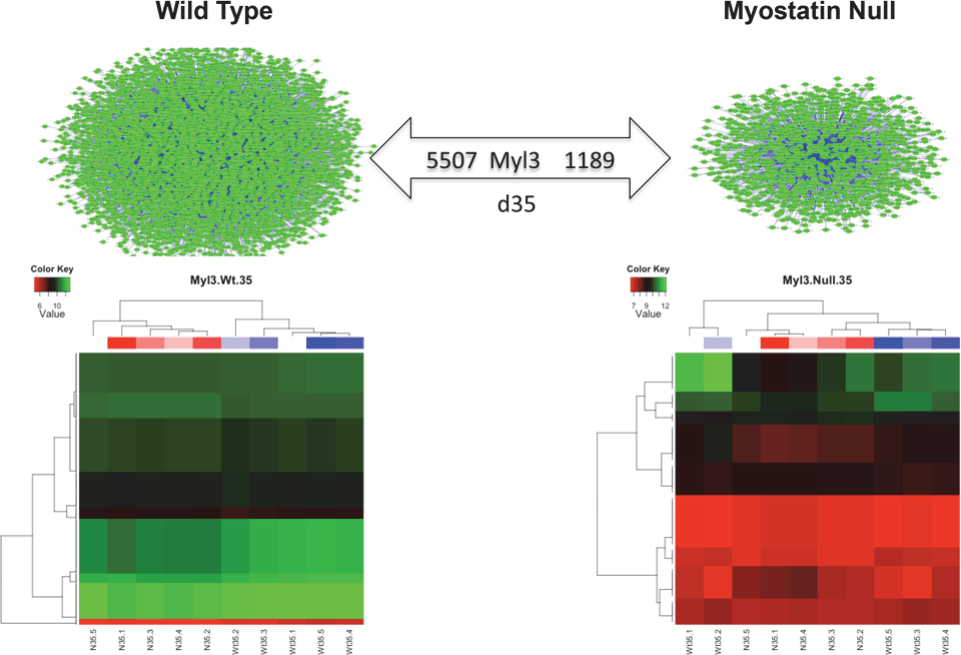
Heat map of genes highly correlated to *Myl3* in WT and MSTN-null mice at d35.

Differential hubbing scores are listed in S6 Table. Fig. 4 illustrates extreme DH observed for *Ctnna1*, *Bmp8b*, and *Myl3* in wild-type vs. MSTN-null mice for highly correlated target genes (|r| ≥ 0.9). For instance, there were over 2,000 genes highly co-expressed (correlated) with *Ctnna1* in MSTN-null mice, but only one gene was highly co-expressed with *Ctnna1* in wild type at 13.5 d.p.c. Interestingly, *Ctnna1* was not differentially expressed (q = 0.56). *Bmp8b* was also found to be differentially hubbed between wild-type and MSTN-null mice at 13.5 d.p.c.; over 3,000 genes were highly co-expressed with *Bmp8b* in wild-type mice, while less than 1,000 genes were highly co-expressed with *Bmp8b* in MSTN-null mice. Fig. 5 contrasts the differences in expression patterns of genes highly correlated to *Myl3* across genotypes at d35. Expression data used in Fig. 5 are listed in S7 Table. The 5,500 genes highly correlated to and co-expressed with *Myl3* in wild-type mice were almost always downregulated, whereas the 1,189 co-expressed genes in MSTN-null mice were almost exclusively upregulated at d35. In MSTN-null mice, the *Myl3* differentially hubbed correlates were enriched for the KEGG pathway term mmu04360: “Axon guidance”. In contrast, those genes highly correlated with *Myl3* in wild-type mice were significantly enriched for the KEGG terms actin cytoskeleton, T/B cell receptor signaling, viral myocarditis, Huntington's disease, cancer pathways, and ubiquitin-mediated proteolysis (Benjamini p < 0.10; Table 7).

**Table 7:** KEGG pathway analysis of genes highly correlated with *Myl3* at d35 in wild-type vs. MSTN-null mice.

Term	Count	%	PValue	List Total	Pop Hits	Pop Total	Fold Enrichment	Benjamini
mmu04810:Regulation of actin cytoskeleton	81	1.82	1.58E-06	1316	217	5738	1.63	2.98E-04
mmu04010:MAPK signaling pathway	93	2.09	5.21E-06	1316	265	5738	1.53	4.92E-04
mmu04662:B cell receptor signaling pathway	37	0.83	9.30E-06	1316	80	5738	2.02	5.86E-04
mmu03010:Ribosome	39	0.88	2.34E-05	1316	89	5738	1.91	1.11E-03
mmu04650:Natural killer cell mediated cytotoxicity	49	1.10	2.93E-05	1316	122	5738	1.75	1.11E-03
mmu04666:Fc gamma R-mediated phagocytosis	41	0.92	5.05E-05	1316	98	5738	1.82	1.59E-03
mmu05340:Primary immunodeficiency	20	0.45	8.99E-05	1316	36	5738	2.42	2.42E-03
mmu04670:Leukocyte transendothelial migration	46	1.03	1.56E-04	1316	119	5738	1.69	3.68E-03
mmu04062:Chemokine signaling pathway	64	1.44	1.78E-04	1316	182	5738	1.53	3.72E-03
mmu04660:T cell receptor signaling pathway	45	1.01	2.66E-04	1316	118	5738	1.66	5.02E-03
mmu04520:Adherens junction	32	0.72	3.52E-04	1316	76	5738	1.84	6.03E-03
mmu04144:Endocytosis	68	1.53	4.54E-04	1316	202	5738	1.47	7.13E-03
mmu05200:Pathways in cancer	100	2.25	5.95E-04	1316	323	5738	1.35	8.61E-03
mmu04142:Lysosome	44	0.99	6.75E-04	1316	119	5738	1.61	9.08E-03
mmu04120:Ubiquitin mediated proteolysis	48	1.08	1.19E-03	1316	136	5738	1.54	1.49E-02
mmu05416:Viral myocarditis	35	0.79	2.33E-03	1316	94	5738	1.62	2.72E-02
mmu04640:Hematopoietic cell lineage	32	0.72	2.49E-03	1316	84	5738	1.66	2.74E-02
mmu04612:Antigen processing and presentation	34	0.76	2.56E-03	1316	91	5738	1.63	2.65E-02
mmu04360:Axon guidance	45	1.01	3.15E-03	1316	131	5738	1.50	3.09E-02
mmu05016:Huntington's disease	58	1.30	5.71E-03	1316	183	5738	1.38	5.27E-02
mmu02010:ABC transporters	19	0.43	7.51E-03	1316	45	5738	1.84	6.56E-02
mmu04514:Cell adhesion molecules (CAMs)	49	1.10	1.05E-02	1316	154	5738	1.39	8.67E-02

## Discussion

### Interpretation of genes differentially expressed during primary myogenesis and postnatal skeletal muscle growth

Microarray analysis of tissues from MSTN-null and wild-type mice identified DE genes at both 13.5 d.p.c. and d35, whereas no DE genes (q < 0.05) were identified at 17.5 d.p.c. Ideally, comparisons would have been made using the same tissue sample from each of the timepoints. However, obtaining single muscles from 13.5 d.p.c. and 17.5 d.p.c. embryos was not feasible due to their small size at these stages of development. An important consequence is that no comparisons can be made across timepoints in this study since the tissue types are not comparable. It is also important to note that results from 13.5 and 17.5 d.p.c. may reflect gene expression changes in tissues other than muscle, even though myostatin is predominantly expressed in muscle. We would also like to note that this study is a meta-analysis of a previous study and that, although there is significant overlap, the number of DE genes identified at d35 in this study differs from the results of the previous study in our lab [13]. This is because different statistical methods were used in these two analyses. Despite the difference in the methodology used to analyze differential expression in the current study, all of the DE genes validated by real-time qPCR in our previous study were also significantly DE in this study (q<0.05; S1 Table) [13].

### Ontology-based enrichment analysis identified biological processes related to myostatin-mediated changes in skeletal muscle growth and metabolism.

DAVID ontology enrichment analyses identified significantly over-represented regulators relevant to both muscle and adipose development. At 13.5 d.p.c., over-representation of the WNT KEGG pathway was identified for genes DE by genotype. This result is consistent with a previous study from our lab that also identified a major role for WNT signal transduction in MSTN-mediated skeletal muscle growth at the d35 postnatal timepoint [13]. The DE genes at d35 were enriched for the DAVID terms muscle organ/tissue development, fatty acid metabolism, and mitochondria, which are all relevant to muscle and adipose growth. All DAVID results are presented in S2 Table.

### MicroRNA-regulated gene networks were over represented at 13.5 d.p.c.

Pathway Studio identified potential regulators within DE gene lists based on text mining of previously published results. Several microRNAs were identified as potential regulators from the sub-network analysis at 13.5 d.p.c., including *Mir29b1*, *Let7g*, *Mir139*, and *Mir122*. Since microRNAs are involved in many early developmental processes, it is possible that they may be involved in muscle and adipose development in pathways upstream or downstream of MSTN signaling.

### Fatty acid metabolism, myogenic, and muscle fiber type pathways were over represented at d35

Pathway enrichment with Pathway Studio at d35 after birth identified a number of significantly enriched pathways (p<0.05) relevant to muscle and adipose development. For example, several myogenic factors’ sub-networks were significantly enriched, including networks regulated by MEF2A, MEF2C, MYH1, and TNNT2 (S3 Table). MEF2C was previously found to be involved in the formation of myofibers [25]. In addition, several sub-networks involved in fatty acid metabolism were also significantly enriched at d35, including networks regulated by ADIPOQ, CEBPA, FABP4, LDL, LEP, PGC1A, PPARG, SREBF1, and VLDL. PPARG, LEP, calmodulin, and GH1 were linked to the largest numbers of differentially expressed genes at d35. UCP2, a mitochondrial membrane transporter involved in energy balance regulation, was significantly enriched as well (p = 0.00573; S3 Table). Notably, WNT4 pathway members were over represented at d35 (p = 0.0227), which is consistent with previously experimentally confirmed results from this study [13]. The transcription factor GATA5, which is an important regulator in muscle development [26], was identified by Pathway Studio because its targets, including the muscle fiber type development genes *Myl2*, *Tnnc1*, *Myh7*, *Myl3*, and *Myh11*, were significantly over represented (p = 0.00133). These results indicate that genes DE due to MSTN loss of function at 13.5 d.p.c. and d35 include several important regulators of muscle and adipose development. All Pathway Studio results are presented in S3 Table.

### Known and novel transcriptional regulatory motifs were differentially enriched in the presence or absence of functional myostatin at 13.5 d.p.c. and d35

Previously, SMAD transcription factors have been implicated in MSTN signal trasnduction [27]; however, not all promoters of MSTN-regulated genes contain SMAD binding sites. Using DREME, several over-represented motifs were identified in DE genes at both 13.5 d.p.c. and d35 (Figs. 1 and 2, Figures A and B in S1 File). Zinc-finger protein family (ZFP) binding site was the most significant motif identified at 13.5 d.p.c. Binding sites for PLAGL1, a known biomarker of disease [28], were over represented in genes upregulated in WT at 13.5 d.p.c. Neither *Plagl1* (q = 0.72) nor *Zfp187* (q = 0.54) was DE at 13.5 d.p.c. However, *Plagl1* itself was significantly 0.67-fold downregulated in WT at d35 (q = 0.01). In contrast, ZFP187 TF binding sites were over represented in genes downregulated in WT at 13.5 d.p.c., while the gene itself was significantly 1.21-fold upregulated in WT at d35 (q = 0.03). It is interesting that PLAGL1 binding sites were identified as over represented in genes that are more highly expressed in the WT genotype, since the similar gene *Plag1* was recently identified in a haplotype that had undergone a selective sweep in double-muscled Belgian Blue cattle [29].

At d35, SMAD3 binding site was found to be the most over-represented motif in DE genes upregulated in MSTN-null mice at d35, indicating its importance in MSTN signal transduction during postnatal growth. SMAD3 has proved to be necessary and sufficient for MSTN signal transduction during muscle development [30]. In addition, SP1 was identified as an over-represented motif in DE genes upregulated in WT at d35. *Sp1* was a DE gene and 1.29-fold upregulated in wild-type mice at d35. The SP1 transcription factor was reported to play an important role in the transcriptional activity of genes [31] and induction of lipogenesis via FOXO1 [32]. Further, SP1 is known to be important in skeletal muscle proliferation through transcriptional regulation of fibroblast growth factor receptor 1 (FGFR1) [33,34], and differentiation by regulation of ERK5/Sp1/Klf [35].

An important unanswered question addressed in this study is how MSTN signals to all of the genes that it regulates. Besides SMAD3 and SP1, additional over-represented motifs were identified in DE genes between MSTN-null and wild-type mice. These results are important because they suggest novel transcriptional regulators that MSTN may signal through to directly or indirectly alter transcriptional regulation of genes within its signal transduction pathway. However, these sequence motifs have no known transcription factors that bind to them. It is possible that currently unknown sequence motifs could regulate MSTN-reactive genes at these sites. These results provide novel insights into possible mechanisms of transcriptional regulation in the context of MSTN signal transduction and indicate that there is still much to learn about MSTN signaling.

### Co-expression methods identified genes known to interact with myostatin as well as novel putative pathway components

Application of the RIF and PIF algorithms [11] to all genes in this dataset (over 34,000 annotated genes) allowed genes to be ranked as regulators, not just transcription factors. This allowed novel regulatory genes to be identified in this study. All PIF scores are provided in S8 Table. Genes with the 20 highest PIF scores at 13.5 d.p.c. (Table 8) were shown by DAVID analysis to be enriched for several RNA and protein modification terms, including protein acetylation, methylation, and RNA-binding (Benjamini p<0.05; S9 Table). Several cell cycle and cancer-related genes were also over represented. The highest PIF value genes from d35 (Table 9) appear to be related to fiber type changes between MSTN-null and wild-type mice (e.g., *Tpm1*, *Ckm*, *Mylpf*, *Actn3*, *Myh4*, *Tnnt3*, *Actc1*, *Myh1*, and *Tnni2*). A DAVID enrichment analysis of the top 20 PIF genes confirms that these genes are involved in a multitude of muscle-specific structural and metabolic functions consistent with fiber type changes that occur due to MSTN inactivation (S9 Table).

**Table 8:** Top 20 PIF at 13.5 d.p.c. *

ProbesetID	PIF Score	13.5 dpc q-value	Gene Symbol	Gene Description
1438009_at	90.96	0.0877	Hist1h2ab, Hist1h2ac, Hist1h2ad, Hist1h2ae, Hist1h2ag, Hist1h2ah, Hist1h2ai, Hist1h2an, Hist1h2ao, Hist1h2ap	histone cluster 1, H2ab, histone cluster 1, H2ac, histone cluster 1, H2ad, histone cluster 1, H2ae, histone cluster 1, H2ag, histone cluster 1, H2ah, histone cluster 1, H2ai, histone cluster 1, H2an, histone cluster 1, H2ao, histone cluster 1, H2ap
1455725_a_at	81.92	0.0527	Gm12657, Gm6749, Gm8029, H3f3a, H3f3b, H3f3c	predicted gene 12657, predicted pseudogene 6749, predicted gene 8029, H3 histone, family 3A, H3 histone, family 3B, H3 histone, family 3C
1420880_a_at	81.02	0.0911	Ywhab	tyrosine 3-monooxygenase/tryptophan 5-monooxygenase activation protein, beta polypeptide
1450849_at	76.60	0.0206	Hnrnpu	heterogeneous nuclear ribonucleoprotein U
1416365_at	75.60	0.0524	Hsp90ab1	heat shock protein 90 alpha (cytosolic), class B member 1
1451285_at	74.64	0.0783	Fus	fusion, derived from t(12;16) malignant liposarcoma (human)
1422848_a_at	72.85	0.0783	Pabpn1	poly(A) binding protein, nuclear 1
1437497_a_at	72.82	0.0295	Hsp90aa1	heat shock protein 90, alpha (cytosolic), class A member 1
1450740_a_at	70.22	0.0308	Mapre1	microtubule-associated protein, RP/EB family, member 1
1423667_at	69.78	0.0788	Mat2a	methionine adenosyltransferase II, alpha
1460339_at	69.52	0.0961	Psma4	proteasome (prosome, macropain) subunit, alpha type 4
1426407_at	68.61	0.0911	Celf1	CUGBP, Elav-like family member 1
1438390_s_at	67.97	0.0444	Pttg1	pituitary tumor-transforming gene 1
1438403_s_at	66.91	0.0487	Malat1	metastasis associated lung adenocarcinoma transcript 1 (non-coding RNA)
1420613_at	66.86	0.0519	Ptp4a2	protein tyrosine phosphatase 4a2
1448425_at	66.77	0.0420	Eif3a	eukaryotic translation initiation factor 3, subunit A
1436884_x_at	66.66	0.0980	Ewsr1	Ewing sarcoma breakpoint region 1
1455283_x_at	65.51	0.0719	Ndufs8	NADH dehydrogenase (ubiquinone) Fe-S protein 8
1453229_s_at	65.50	0.0914	Uqcrh	ubiquinol-cytochrome c reductase hinge protein
1455546_s_at	65.10	0.0652	Sf3a2	splicing factor 3a, subunit 2

* The top 20 PIF scores with a q-value < 0.10 are presented. For multiple probesetIDs with the same annotation, the probesetID with smallest q-value was saved.

**Table 9:** Top 20 PIF at d35*.

ProbesetID	PIF Score	d35 q-value	Gene Symbol	Gene Description
1423049_a_at	105.15	0.0584	Tpm1	tropomyosin 1, alpha
1417614_at	104.42	0.0645	Ckm	creatine kinase, muscle
1417653_at	101.76	0.0538	Pvalb	parvalbumin
1448371_at	101.03	0.0230	Mylpf	myosin light chain, phosphorylatable, fast skeletal muscle
1418677_at	100.56	0.0022	Actn3	actinin alpha 3
1427026_at	98.24	0.0298	Myh4	myosin, heavy polypeptide 4, skeletal muscle
1417951_at	96.84	0.0594	Eno3	enolase 3, beta muscle
1455736_at	96.20	0.0047	Mybpc2	myosin binding protein C, fast-type
1456588_x_at	96.03	0.0271	Cox5b LOC100046079	cytochrome c oxidase, subunit Vb, cytochrome c oxidase, subunit 5B, mitochondrial-like
1450118_a_at	94.88	0.0517	Tnnt3	troponin T3, skeletal, fast
1418373_at	94.26	0.0283	Pgam2	phosphoglycerate mutase 2
1415927_at	93.14	0.0122	Actc1	actin, alpha, cardiac muscle 1
1416624_a_at	92.19	0.0524	2810422J05Rik Uba52	RIKEN cDNA 2810422J05 gene, ubiquitin A-52 residue ribosomal protein fusion product 1
1417373_a_at	91.83	0.0255	Tuba4a	tubulin, alpha 4A
1419737_a_at	91.42	0.0102	Ldha	lactate dehydrogenase A
1418062_at	91.42	0.0121	Eef1a2	eukaryotic translation elongation factor 1 alpha 2
1460561_x_at	90.62	0.0041	Sepw1	selenoprotein W, muscle 1
1427868_x_at	90.48	0.0089	Myh1	myosin, heavy polypeptide 1, skeletal muscle, adult
1426143_at	90.05	0.0358	Trdn	triadin
1438609_x_at	90.02	0.0819	Tnni2	troponin I, skeletal, fast 2

*The top 20 PIF scores with a q-value < 0.10 are presented. For multiple probesetIDs with the same annotation, the probesetID with smallest q-value was saved.

Several known regulators of skeletal muscle growth were identified using the RIF method. Potentially novel regulators that fit closely with known MSTN functions were also identified. The following passages present several novel possibilities regarding regulation of MSTN signal transduction.

At 13.5 d.p.c., voltage-dependent anion channel 1 (*Vdac1*) and thymosin B4 (*Tmsb4x*) were identified among the top ten RIF2 scores. The *Vdac1* gene encodes a mitochondrial porin that appears to regulate mitochondrial function by regulating the metabolites and ions that can enter the mitochondria [36,37]. A recent study reports that treatment of HeLa cells with MSTN leads to increases in VDAC1-mediated apoptosis via BAX [38]. Interestingly, *Vdac1* knockout mice also have impaired exercise capacity and reduced glucose tolerance [36]. The *Tmsb4x* gene has been proposed to be involved in wound healing and muscle regeneration. Studies in C2C12 and satellite cell–derived primary myocytes and myoblasts indicate that *Tmsb4x* is more highly expressed at muscle wound sites and that it acts as a myoblast chemoattractant during muscle regeneration [39]. In the mdx mouse model of muscular dystrophy, chronic administration of TMSB4X was also found to increase the number of regenerating skeletal muscle fibers [40].

The *Sox2* gene was identified as a top-ten ranked RIF1 gene at 13.5 d.p.c. Several studies have documented the role of SOX2 in SMAD-mediated TGFβ signal transduction during embryonic development [41]. In addition, *Sox2* sustained expression is promoted by TGFβ signal transduction to promote embryonic stem cell self renewal [42]. Therefore, it is plausible that altered MSTN signal transduction, which acts through TGFβ, may impact SOX2 signal transduction and processes associated with stem cell renewal. Since whole embryos were used for 13.5 d.p.c., it is not possible to determine which tissues are actually affected.

At d35, *Apobec2* and *Mmp13* were identified as potential regulators based on high RIF2 and RIF1 scores, respectively. The APOBEC2 protein is an RNA-editing enzyme that is expressed almost exclusively in striated muscle and appears to be important in fiber type determination. Mouse knockouts of *Apobec2* tend to have a greater ratio of slow to fast twitch muscle, 15-20% reduction in body mass, and evidence of mild myopathy in aged mice [43]. Vonica et al. have defined the specific roles of APOBEC2 in right/left axis patterning in development, terminal differentiation of muscle, and inhibition of TGFβ signal transduction [44]. Based on studies in zebrafish, it has been suggested that APOBEC2 may mediate its effects in part by altering methylation patterns [44,45]. The role of MMP13 in skeletal muscle has been suggested to center around myoblast migration and differentiation during muscle regeneration. Pharmacological blockade of MMP13 in C2C12 cells leads to reduced myoblast migration and differentiation, whereas overexpression leads to improved myoblast migration. Expression of *Mmp13* appears to be increased during muscle repair as well as following myoblast fusion during myotube formation. Although its exact role is unknown, it has been suggested to alter extracellular matrix remodeling during muscle regeneration [46].

The identification of *Atp2a2* (a.k.a. *Serca2a*) as the top-ranked RIF2 gene at d35 is particularly noteworthy, because it was previously identified as a potential modifier of MSTN in a large F2 mouse study [47]. The ATP2A2 calcium pump affects sarcoplasmic reticulum function and muscle fiber type. Transgenic *Atp2a2* mice exhibit cardiac hypertrophy [47–50]. Furthermore, pharmacological inhibition of SMAD2 in cardiomyocytes indicates that TGFβ/SMAD2 signaling regulates ATP2A2 function [51]. Therefore, it is very plausible that MSTN may regulate ATP2A2 in skeletal muscle to mediate changes in muscle fiber type and size.

Differentially hubbed genes that may act as regulators or key mediators in muscle and adipose development in the context of MSTN are listed in S1 Table. *Ctnna1* was identified as differentially hubbed at 13.5 d.p.c. despite not being DE, which indicates that MSTN may affect some regulators both directly and indirectly so as to cause or influence the differential expression of target genes. Similarly, *Bmp8b* was identified to have over 2,000 genes differentially hubbed in wild-type versus MSTN-null mice (Fig. 4). A recent report demonstrated that BMP8B directly regulates thermogenesis in brown adipose tissues [52], which indicates that MSTN may alter adipose metabolism through BMP8B. In addition, recent research identified a mutation in *Myl3* that can cause hypertrophic cardiomyopathy in infants [53]. In our study, over 4,000 genes were differentially hubbed with *Myl3* in wild-type compared to MSTN-null mice. Interestingly, significant enrichment of biological pathways involved in muscle development and disease occurred only in wild-type mice (Table 7, adj.P, Benjamini < 0.1), which is consistent with previous findings that MSTN plays an important role in phenotypes such as muscle hypertrophy or disease caused by muscle dysfunction [54,55].

## Conclusions

Differentially expressed genes were identified between wild-type and MSTN-null mice during primary myogenesis and postnatal muscle growth, but not during secondary myogenesis. In addition, co-expression analyses identified several known and novel regulators or pathway effectors of myostatin. Regulators with known roles in muscle growth, TGFβ signaling, and mitochondrial function were identified by the RIF method. Another important finding was that genes not DE or a priori considered regulators were identified as important in differentiating wild-type and MSTN-null mice. These potential regulators would not have been identified if analyses had been limited to considering only transcription factors as regulators. Furthermore, motif enrichment analyses identified several transcription factor binding sites in genes differentially expressed due to Mstn genotype. This study demonstrates that all genes must be considered as potential regulators when trying to identify critical control points of pathway circuitry.

## Supporting Information

S1 File Supporting figures. Figure A in S1 File. Over-represented motifs in genes upregulated in WT at d35. Figure B in S1 File. Over-represented motifs in genes upregulated in MSTN-null at d35.
(PDF)

S1 Table Results of microarray analysis. (XLSX)

S2 Table Results of DAVID analysis from DE genes at 13.5 d.p.c. and d35. (XLSX)

S3 Table Results of pathway analysis from DE genes at 13.5 d.p.c. and d35. (XLSX)

S4 Table Results of the RIF analysis (Zscore). (XLSX)

S5 Table Over-represented KEGG terms for genes highly correlated with *Tnni2* at d35. (XLSX)

S6 Table Results of differential hubbing. (XLSX)

S7 Table Data for Fig. 5. (XLSX)

S8 Table Results of the PIF analysis. (XLSX)

S9 Table DAVID enrichment of extreme PIF values. (XLSX)
