# Supplementary material for: Gene Co-Expression Network Analysis Provides Novel Insights into Myostatin Regulation at Three Different Mouse Developmental Timepoints
Source: PLoS One. 2015 Feb 19;10(2):e0117607. doi: 10.1371/journal.pone.0117607 (PMC4335066; doi:10.1371/journal.pone.0117607)
Supplement: S1 File — Over-represented motifs in genes upregulated in WT at d35. Figure B in S1 File. Over-represented motifs in genes upregulated in MSTN-null at d35. (PDF) [file pone.0117607.s001.pdf]

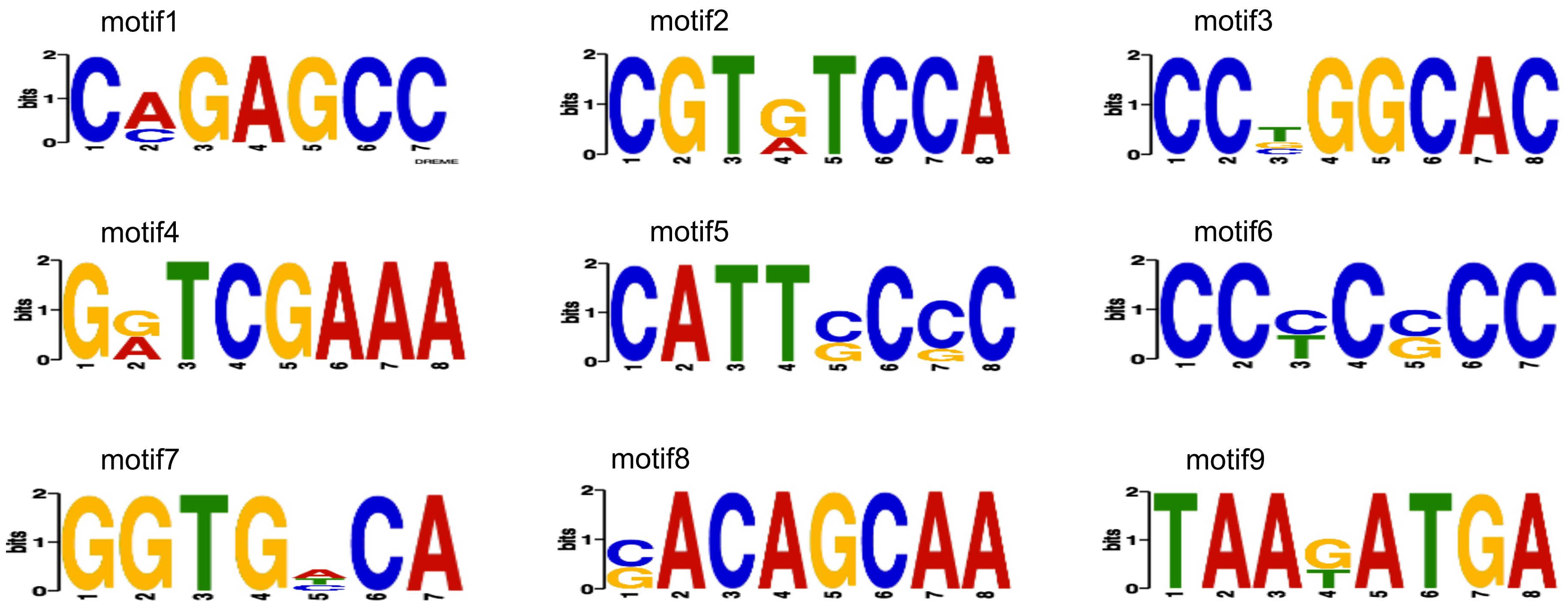

Motif#	Sequence(IUPAC)	TOMTOM
1	CMGAGCC	
2	CGTRTCCA	
3	CCBGGCAC	
4	GRTCGAAA	
5	CATTSCSC	
6	CCYCSCC	Zfp281_primary, SP1
7	GGTGHCA	
8	SACAGCAA	
9	TAAKATGA	

Over-represented motifs in genes upregulated by myostatin on d35

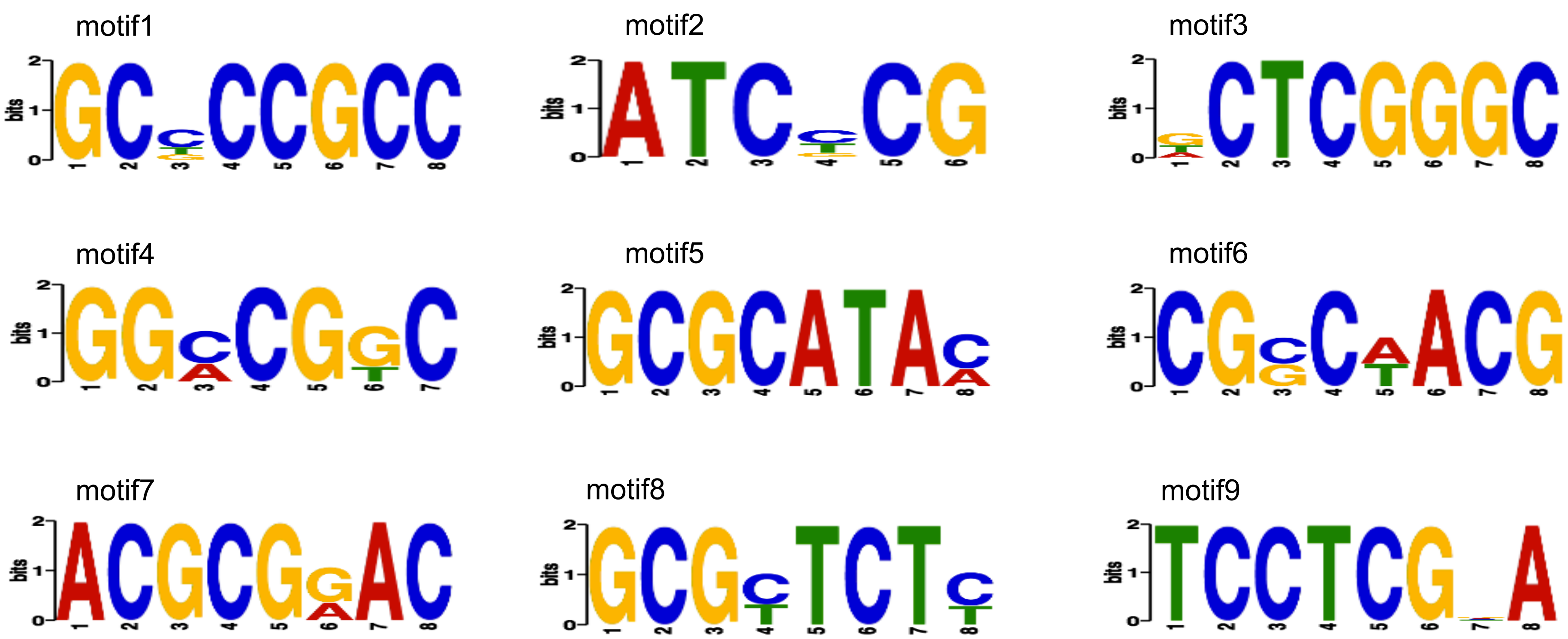

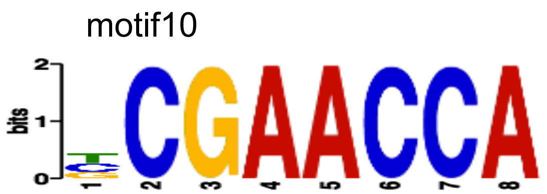

Motif#	Sequence(IUPAC)	TOMTOM
1	GCBCCGCC	Smad3_secondary, CHA4, Klf4
2	ATCBCG	
3	DCTCGGGC	
4	GGMCGKC	
5	GCGCATAM	
6	CGSCWACG	
7	ACGCGRAC	
8	GCGYTCTY	
9	TCCTCGNA	
10	BCGAACCA	

Over-represented motifs in genes downregulated by myostatin on d35
